# A Modular Cooperative Wall-Climbing Robot Based on Internal Soft Bone

**DOI:** 10.3390/s21227538

**Published:** 2021-11-12

**Authors:** Wenkai Huang, Wei Hu, Tao Zou, Junlong Xiao, Puwei Lu, Hongquan Li

**Affiliations:** School of Mechanical and Electrical Engineering, Guangzhou University, Guangzhou 510006, China; smallkat@gzhu.edu.cn (W.H.); 1707700083@e.gzhu.edu.cn (W.H.); 1707200071@e.gzhu.edu.cn (J.X.); 2111907042@e.gzhu.edu.cn (P.L.); 1807300127@e.gzhu.edu.cn (H.L.)

**Keywords:** wall-climbing robot, modular, variable step distance, variable load, internal soft bone, payload power factor

## Abstract

Most existing wall-climbing robots have a fixed range of load capacity and a step distance that is small and mostly immutable. It is therefore difficult for them to adapt to a discontinuous wall with particularly large gaps. Based on a modular design and inspired by leech peristalsis and internal soft-bone connection, a bionic crawling modular wall-climbing robot is proposed in this paper. The robot demonstrates the ability to handle variable load characteristics by carrying different numbers of modules. Multiple motion modules are coupled with the internal soft bone so that they work together, giving the robot variable-step-distance functionality. This paper establishes the robotic kinematics model, presents the finite element simulation analysis of the model, and introduces the design of the multi-module cooperative-motion method. Our experiments show that the advantage of variable step distance allows the robot not only to quickly climb and turn on walls, but also to cross discontinuous walls. The maximum climbing step distance of the robot can reach 3.6 times the length of the module and can span a discontinuous wall with a space of 150 mm; the load capacity increases with the number of modules in series. The maximum load that N modules can carry is about 1.3 times the self-weight.

## 1. Introduction

Wall-climbing robots have attracted great interest from researchers because of their potential application value, including in building and ship inspection, materials transportation, search and rescue, and other tasks [1]. Generally, wall-climbing robots need to be able to carry a variety of sensors or transport required materials; therefore, load capacity is an important performance index for these robots. A variable load capacity renders the robot more adaptable to tasks. In addition, when such a robot faces a discontinuous wall with particularly large spaces, the ability to adjust its step distance and use a larger step allows a wall-climbing robot to adapt to complex environments. Therefore, studying variable loads and variable step distances with wall-climbing robots is highly pertinent.

Many scholars have tried to improve the load capacity of wall-climbing robots. The wall-climbing robots proposed earlier have mainly been cleaning robots [2,3,4,5,6]. Zhang et al. [3] proposed the Sky Cleaner 3 robot, which is a relatively mature wall-climbing cleaning robot based on suction-cup adsorption. The robot can carry about 60 kg of payload, including its own weight (45 kg). Lee’s team [7] developed a series of multilinked caterpillar track (MCT)-type climbing robots with different objectives. The robots range from small (180 g) to large (70 kg), while payloads range from 0.5 kg to 15 kg. Huang et al. [8] introduced a crawler wall-climbing robot using magnetic adsorption for ship detection. The payload of the robot is 6 kg and has strong adaptability to the ship environment. Eto et al. [9] proposed a new wheeled wall-climbing robot, which also relies on magnetic attachment to the ferromagnetic wall for complex welding of metal hull. The robot weighs 7.4 kg and can carry 4 kg of welding tools. A detection robot capable of climbing concrete structures has been proposed by Garrido et al. [10]. It relies on permanent magnet absorption and wheel drive, which makes it highly loadable. The above-mentioned wall-climbing robots using vacuum and magnetic adsorption as their adsorption principle have relatively strong load capacity; however, the author found that this capacity is usually related to the size and weight of the robot itself; that is, if you want to increase their load capacity, you need to add more hardware equipment yourself. This can meet load demand, but it increases the complexity of self-control and the risks of operation. A modular wall-climbing robot can share the load among its own modules, and by slightly increasing the complexity of the machine, its load capacity can be greatly improved.

Climbing robots have to be provided with a proper locomotion and adhesion system with respect to the surface they have to climb [11]. The advantages and disadvantages of different ways of moving and sticking have been studied in detail by some researchers [11,12]. However, the increasingly complex designs of wall-climbing robots entail new requirements for terrain–environment adaptability. For complex wall climbing, wall-climbing robots relying on foot motion [13,14,15,16,17,18,19] generally have higher degrees of freedom and have higher adaptability to the environment than wheeled and crawler wall-climbing robots. Guan et al. [18] proposed a wall-climbing robot with bipedal motion. Its unique inchworm motion enables it to move on discontinuous discrete surfaces with high flexibility. The Hexapod wall-climbing robot designed by Gao et al. [14] can span different walls. Bionic wall-climbing robots using peristaltic, inchworm, crawling, and other motion modes [1,20,21,22,23,24,25] can also move on complex walls by adapting to rough, uneven, and irregular contact surfaces. Although the above-mentioned wall-climbing robots have strong adaptability to continuous climbing environments, in each case, their movement ability is restricted to small steps, and it remains a challenge for these robots to cross discontinuous contact surfaces with very large spaces. When a space is close to or larger than the length of the mobile unit of the robot, a single robot cannot cross, whereas a modular wall-climbing robot with a large step distance has the potential to do so.

Therefore, based on previous research, this paper proposes a bionic crawling modular wall-climbing robot based on internal soft bone (ISB-MWCR), which is used to improve load capacity and to span large gaps in discontinuous contact surfaces. The robot can respond to variable load characteristics by carrying different numbers of modules to increase its payload range. It can also manifest the functionality of variable step distance via the cooperative operation of multiple motion modules and internal soft bones to span large spaces in a discontinuous wall.

The main contributions of this paper are as follows:

By analyzing the movement mode of the leech, a bionic crawling modular wall-climbing robot based on internal soft bone was designed. The motion planning for the robot is presented, and the internal soft-bone and multiple-motion modules are coupled in series to enable flexible climbing, steering, and spanning motions. The kinematics analysis and finite element simulation of the robot module are also presented.

The climbing, steering, space-spanning, and load-movement experiments on the modular climbing robot are described. Our experiments show that the modular climbing robot can climb quickly and turn on smooth and flat walls. The load capacity increases with the number of modules in series. The maximum load that N modules can carry is about 1.3 times the self-weight. On the premise of stable movement, the mobile walking distance of the robot can reach up to 3.6 times the length of the module. Furthermore, it can span a discontinuous wall with 150 mm spacing, and the effective variable step distance is 0 mm to 400 mm. In addition, we also propose a performance index for the load performance of the ISB-MWCR, which is called the payload power factor. The maximum payload power factor of the robot module is 0.582.

## 2. Materials and Methods

### 2.1. Robot Structure Design

**Figure 1:**
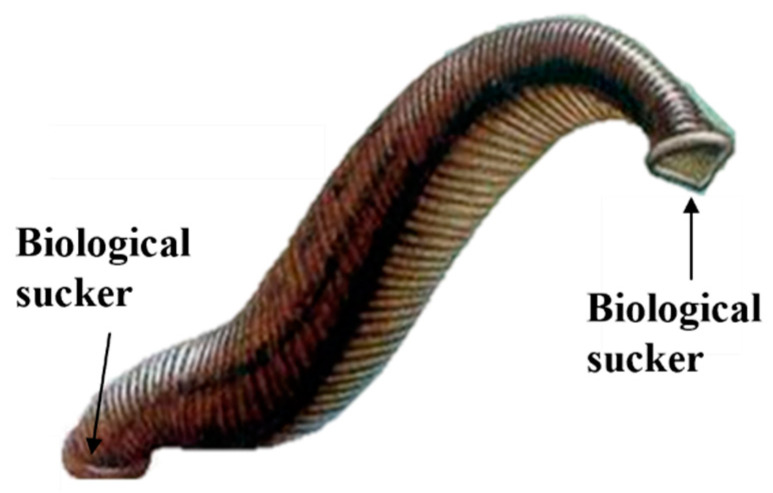
A leech.

The wonders of the natural world are a constant source of inspiration. Through the observation of leeches (Figure 1), it is not difficult to see that they are able to move about in water and on land only by use of their suction cups and muscles. In the water, they spread out their bodies and perform wave swimming by stretching their muscles. On land, they can perform peristaltic or inchworm motion [21].

**Figure 2:**
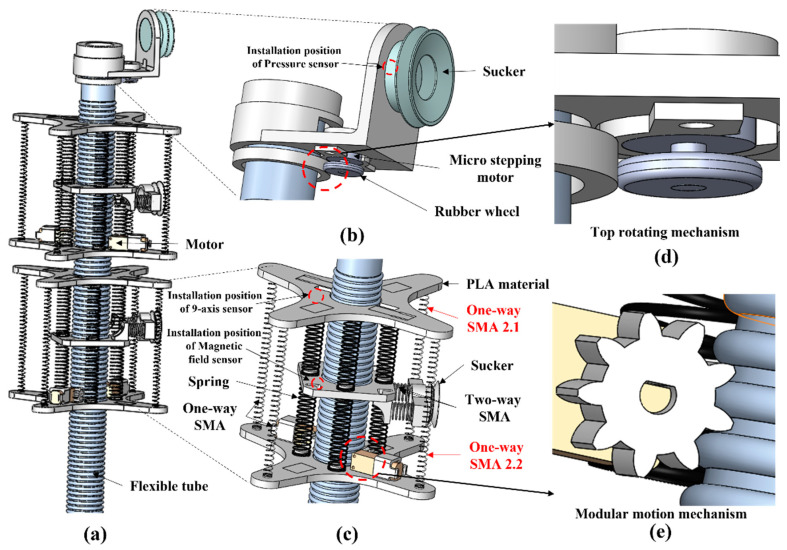
Schematic diagram of ISB-MWCR. (**a**) Overview. (**b**) Rotary adsorption structure of robot head. (**c**) Modular unit of robot. (**d**) Top rotating mechanism. (**e**) Modular motion mechanism.

Inspired by and based on the unique peristaltic mode of the leech, this paper presents the design of an ISB-MWCR (Figure 2a provides an overview). As shown in the figure, which presents two modules for the purposes of example, the entire robot is composed of two module units: internal soft bone, and rotary adsorption mechanisms on the top of the internal soft bone. The structure of the rotary adsorption mechanism is shown in Figure 2b,d. It simulates the functions of biological suction cup adsorption and muscle torsion of a leech head through a suction cup and a micro-stepping motor. The internal soft bone of the robot and the module units connected in series (as shown in Figure 2c,e) simulate the functions of the body muscles and tail suckers of leeches. The relative motion between the module unit and the internal soft bone is generated through the meshing transmission of the polylactic acid (PLA) gear, driven by the reduction motor on the module and the internal soft bone, as well as the alternating adsorption of the suction cups on the top of the module and the internal soft bone. The one-way shape-memory alloy (SMA) on the module unit can bend the module and internal soft bone in a way that mirrors the torsion of leech body muscles. The degrees of freedom of this robot have the following relationship: Dof=3N+1, where N is the number of modules.

### 2.2. Motion Planning

The movement mode of the robot resembles that of many crawling creatures in nature, especially leeches, with their biological suction cups at the head and tail. They all carry out the process of repeated extension/contraction, grasping/releasing, and offset/correction of the body. Therefore, we planned two basic motions for the robot, namely, climbing motion and steering motion, taking two module units in series as an example.

**Figure 3:**
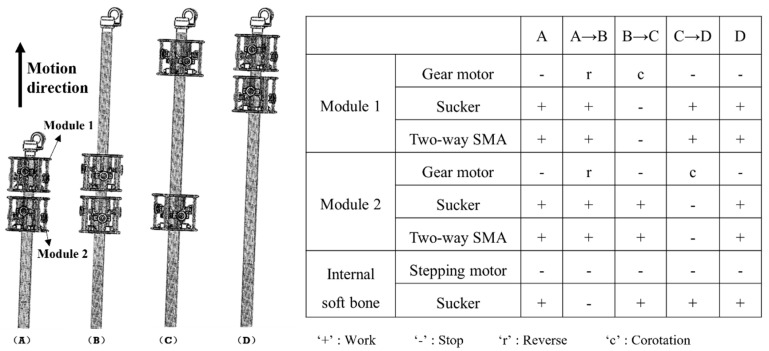
Climbing motion planning of ISB-MWCR and the status of its corresponding components.

The climbing movement is shown in Figure 3. The left side of the figure shows the schematic diagram of one cycle and four periods of the robot’s climbing movement, and the right side shows the state of each functional component of the robot in five stages. ‘A–D’ refer to the four periods of the robot, ‘+’ represents the working state of the parts, ‘-’ represents the non-working state of the parts, ‘r’ represents the reverse rotation of the deceleration/stepping motor, and ‘c’ represents the forward rotation of the deceleration/stepping motor. When the component is in the working state, the suction cup is in the adsorption state, the two-way SMA is in the power-on extension state, and the one-way SMA is in the power-on contraction state. When the component is in the non-working state, the suction cup does not adsorb, while the two-way SMA is in the state of power-off contraction, and the one-way SMA is in the power-off state and stretched by external force.

**Figure 4:**
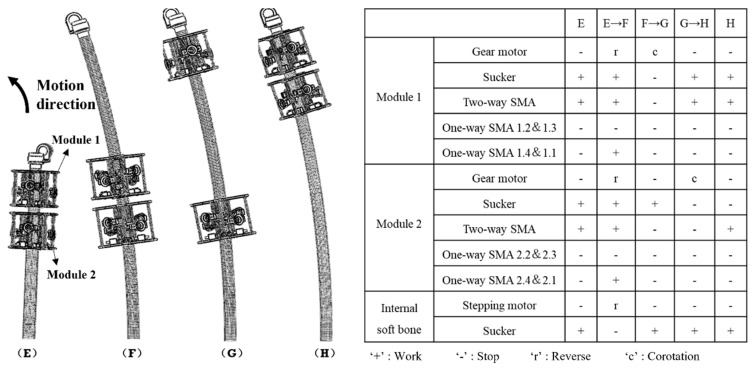
Steering motion planning of ISB-MWCR and the status of its corresponding components.

The steering movement is shown in Figure 4, which presents steering movement to the left as an example. In the table on the right side of Figure 4, ‘1.1’ or ‘1.2’ represent the number of one-way SMAs; for example, ‘2.1’ represents the one-way SMA at the first position of the second module of the robot, as marked in Figure 2c. The step length of the climbing movement of the entire robot or module unit is controlled and adjusted by the working time of the reduction motor and the data fed back by the displacement sensor. The speed of the reduction motor is known, and the adsorption state of the suction cup is fed back by the embedded pressure sensor. The steering movement adds the contraction of the one-way SMA based on the climbing movement, which directly leads to the bending of the module unit to indirectly bend the internal soft bone, thus realizing the steering movement. The steering deflection is controlled and adjusted by the working time of the one-way SMA and the data fed back by the angle sensor.

### 2.3. Kinematics Analysis and Simulation

#### 2.3.1. Kinematics Analysis

By studying the creeping of a leech on a two-dimensional plane, this paper presents two forms of ISB-MWCR plane motion: one is climbing motion and the other is steering motion. The former only needs mutual movement between the module unit and the internal soft bone for climbing, while the latter also adds the bending function of the module unit based on climbing. To provide a theoretical basis for robot motion, kinematics modeling and analysis of robot climbing and steering was carried out.

**Figure 5:**
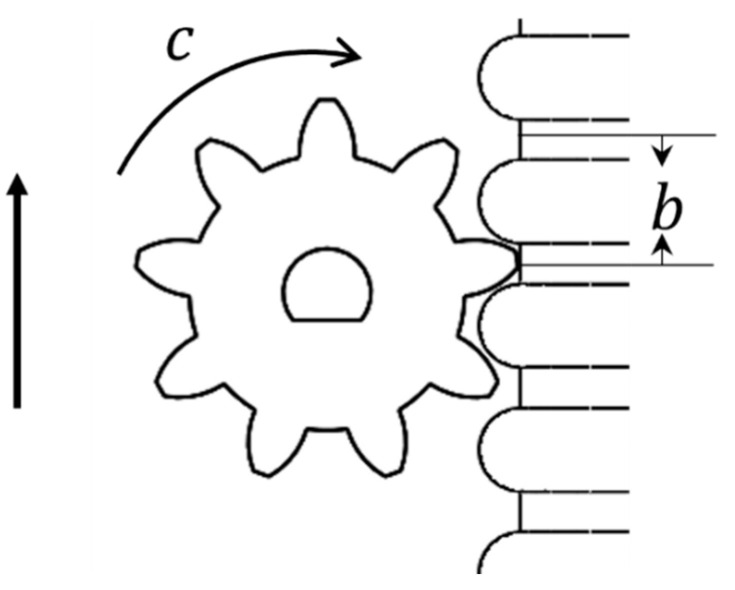
Modular climbing motion model.

Climbing kinematics: the robot generates climbing motion by relying on the reduction motor on the module unit and driving along the internal soft-bone guide rail by means of gears. Accordingly, the climbing motion can be simplified into the model shown in Figure 5.

The model accords with the gear transmission law:

(1) S=ctbn

where S is the movement distance on the internal soft-bone guide rail, c is the speed of the DC motor, t is the working time, b is the gear tooth pitch, and n is the number of gear teeth.

Steering kinematics: the robot bends the module itself and the internal soft bones through the contraction of the one-way SMA on the module unit to deflect the top of the robot. Therefore, we scale the steering motion of the robot to the bending motion of the module for kinematic modeling and analysis.

**Figure 6:**
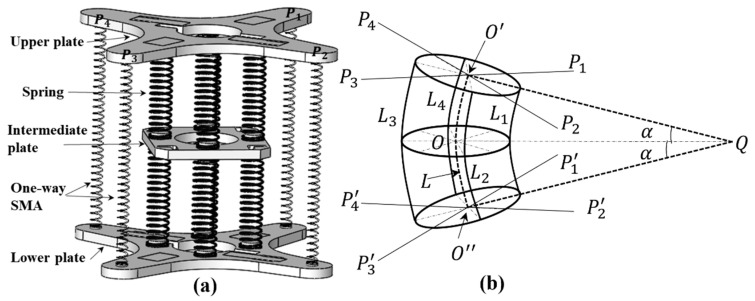
Three-dimensional simplified model of the module. (**a**) 3D simplified solid model of the module. (**b**) Geometric model of module element in bending.

**Figure 7:**
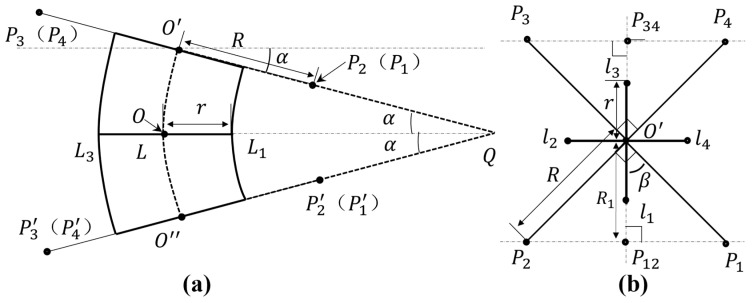
Two-dimensional model of the module. (**a**) A two-dimensional bending model of the module projected on Reference Plane 1. (**b**) Top view of the upper plate plane when the module is not bent.

The three-dimensional simplified model of the module unit is shown in Figure 6. $L_{1}$, $L_{2}$, $L_{3},$ and $L_{4}$ represent eight springs connecting three plates, which are simplified to four in this model. The central axis of the module is marked as L, and $O^{\prime };\;O$, and $O^{\prime \prime }$ are the intersections of the three faces and the central axis, respectively. $P_{i}$ and $P_{i}^{\prime }\left(i=1,\;2,\;3,\;4\right)$ are the connection points between each one-way SMA in the module and the upper and lower plates, respectively; the line connected with the central axis is the distance from these to the central axis, and they are all equal. The included angle between the upper plate plane, the lower plate plane, and the middle plate plane due to module bending is marked as α. Q is the intersection of two plane extension lines. When the module is bent, the force generated by the contraction and tension of one-way SMA is bidirectional, and the positions and specifications of the springs installed above and below are consistent. Therefore, the tensile force on the upper plate and the lower plate during the contraction and tension of one-way SMA can be approximately equal; that is, the bending kinematics of the upper and lower modules of the middle plate have a symmetrical relationship. Take the plane of angle α and springs $L_{1}$ and $L_{3}$ as the reference plane, which is defined as Reference Plane 1. If the three-dimensional model of Figure 6 is projected onto Reference Plane 1, the two-dimensional bending model of the module can be represented as shown in Figure 7a.

In Figure 7a, due to projection, the springs $L_{2}$ and $L_{4}$ coincide with the central axis, which is not shown in the figure. The distance from the contact point between each one-way SMA and the upper and lower plates to the central axis is R, such as $O^{\prime }P_{2}=R$. The nearest distance from each spring to the central axis is r, such as the distance from spring $L_{1}$ to point O of the central axis. In Figure 7b, $l_{i}\;\left(i=1,\;2,\;3,\;4\right)$ is the connection point between each spring and the upper plate; that is, the distance from these four points to point $O^{\prime }$ is r. Taking the straight line where $O^{\prime }l_{1}$ is located as the reference, the projection of $P_{1}$ and $P_{2}$ is $P_{12}$, the projection of $P_{3}$ and $P_{4}$ is $P_{34}$, ∠$P_{2}OP_{1}$ and ∠$P_{3}OP_{4}$ are right angles, and ∠β=45°, yielding the following geometric relationship:

(2) $$l_{OP_{12}}=l_{OP_{34}}=R_{1}=\frac{\sqrt{2}}{2}R$$

**Figure 8:**
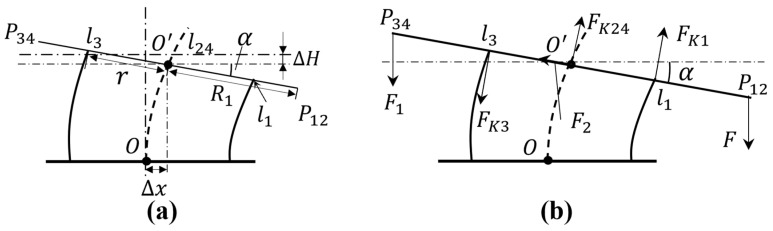
Analysis of the bending movement of the upper half of the module. (**a**) Analysis of the posture change of the upper part when the module is bent. (**b**) Analysis of the plane stress of the upper plate when the module is bent.

Because the frame structure of the module unit is symmetrically designed, after simplifying the model, the upper and lower parts of the module unit can be taken as exhibiting approximately mirror motion when the module is bent. Therefore, the upper half of the module will be taken as an example for bending kinematics analysis. As shown in Figure 8a, when the upper half of the module is bent to the right, the motion can be regarded as: (1) the distance of the upper plate plane moving downward ΔH [26], (2) then moving to the right Δx, and (3) finally generating a bending motion with angle α. Therefore, the amount of change in the vertical direction when each point module is bent is as follows:

(3) $$\{\begin{array}{c} \;P_{12}:\;\Delta L_{S12}=R_{1}sin\alpha +\Delta H\\ \;P_{34}:\;\Delta L_{S34}=R_{1}sin\alpha -\Delta H\\ l_{1}:\;\Delta L_{P1}=rsin\alpha +\Delta H\\ \;l_{3}:\;\Delta L_{P3}=rsin\alpha -\Delta H\\ l_{24\;}:\;\Delta L_{P24}=\Delta H \end{array}$$

In Equation (3), point $l_{24}$ represents the projection point of $l_{2}$ and $l_{4}$ on Reference Plane 1, which coincides with point $O^{\prime }$ in Figure 8a. $\Delta L_{S12}$ represents the displacement change of $\;P_{12}$.

In the stress analysis diagram of Figure 8b, F is the resultant force generated against the upper plate when two one-way SMAs contract. Due to the object characteristics and installation mode of one-way SMA, the direction of resultant force F is always vertically downward. $F_{1}$ is the reaction force obtained by stretching two one-way SMAs, and the direction is also always vertically downward.$\;F_{2}$ is the reaction force received by the module when bending the internal soft-bone, and the direction is upward along the plane of the upper plate.$\;F_{K1}$ and $F_{K3}$ are the reaction forces of compression and tension of springs $L_{1}$ and $L_{3}$ between the upper plate and the middle plate, respectively, and the direction is always perpendicular to the plane of the upper plate.$\;F_{K24}$ is the compression reaction force of springs $L_{2}$ and $L_{4}$ between the upper plate and the middle plate (i.e., $F_{K24}=F_{K2}+F_{K4}$), and the direction is always perpendicular to the plane of the upper plate.

According to the force balance, the direction of the projection line perpendicular to the upper plate plane has the following relationship:

(4) $$\left(F+F_{1}\right)cos\alpha +F_{K3}=F_{K24}+F_{K1}$$

The directions along the projection line of the upper plate plane are

(5) $$\left(F+F_{1}\right)sin\alpha =F_{2}$$

According to Hooke’s Law:

(6) $$F_{Ki}=k\Delta L_{pi,\;}\;i=1,\;2,\;3,\;4$$

In Equation (6), k is the spring coefficient of the selected spring, and $\Delta L_{i\;}$ is the change in the vertical direction of each spring, for which:

(7) $$\Delta L_{P2}=\Delta L_{P4}=\Delta L_{P24}$$

By assuming torque balance for point $O^{\prime }$ in Figure 8b, where clockwise is positive, the following is obtained:

(8) $$FR_{1}cos\alpha -F_{1}R_{1}cos\alpha -F_{K1}r-F_{K3}r=0$$

Simultaneous Equations (4) and (8) are then

(9) $$\{\begin{array}{c} \left(F+F_{1}\right)cos\alpha +F_{K3}=F_{K24}+F_{K1}\\ FR_{1}cos\alpha -F_{1}R_{1}cos\alpha -F_{K1}r-F_{K3}r=0 \end{array}$$

Equation (9) is the kinematic model of the robot module when bending. The closing force F belongs to the main force, and its size is related to the voltage and time required to energize the SMA. The reaction force $F_{1}$ is a passive force, and its size is related to the tensile properties of the SMA. In Section 3.2 of this paper, we will test the SMA used by the robot and obtain expressions of F and $F_{1}$.

#### 2.3.2. Motion Simulation

**Figure 9:**
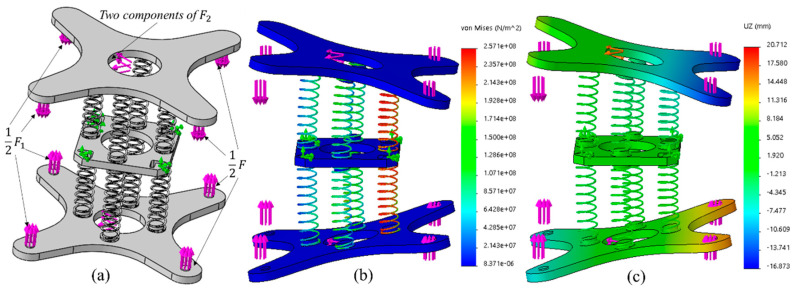
(**a**) Load application. (**b**) The stress distribution on the module element during bending simulation. (**c**) Displacement and deformation.

To better understand the bending motion of the robot based on the established kinematics model, finite element simulation analysis of the module unit of the robot was conducted using the simulation module of SolidWorks. To simplify the analysis process, the one-way SMA is replaced by load in the simulation model, and other components not related to the simulation are omitted, as shown in Figure 9a. Firstly, when defining the material, the composition of the spring is defined as 65 Mn high-quality carbon structural steel, and that of the upper, middle, and lower plates is defined as ABS plastic. Then, the joint connection mode in global contact is adopted between parts. Next, the load is defined. In practice, the middle plate is relatively fixed, so the middle plate is fixed in the analysis. The effect of one-way SMA and internal soft bone is replaced by direct force according to the force analysis in Figure 8b. The application of each force during the simulation is shown in Figure 9a. The values of forces F, $F_{1}$, and $F_{2}$ can be taken according to the test of materials, F=10 N, $F_{1}=3.6\;N$, $F_{2}=4\;N$. Finally, the grid is generated and analyzed. The simulation results of stress and displacement are shown in Figure 9. From the simulation results, we can see the bending of the module. At the two end points on the right side of the upper plate, the maximum displacement is about 16.8 mm, while the result of the kinematic model is 19.4 mm, the difference between them is 2.6 mm, and the error is about 13.4%. This error may be caused by the nonlinear deformation of the spring.

## 3. Experimental Results

### 3.1. Hardware System Design

**Figure 10:**
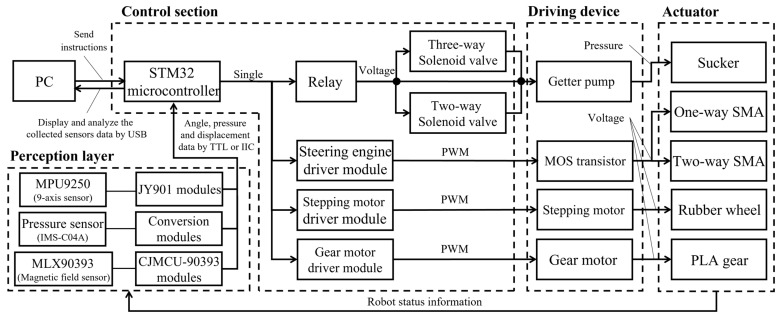
Control system hierarchy of the ISB-MWCR.

Before starting the climbing experiment of the ISB-MWCR, to enable its motion function, we designed a robot control system for the robot, as shown in Figure 10. The system is mainly divided into four parts: the control section, the driving device, the actuator and perception layer, and its control core, which is an STM32 microcontroller. This study uses a USB connection between the microprocessor and a PC to send and receive control instructions. Then, the sensors and various control modules are controlled and connected through the signal port of the microprocessor to receive data and transmit control instructions. The corresponding driving device is controlled by various driving modules, and finally, the actuator is activated. While the actuator is operating, the sensing layer composed of various sensors feeds the robot’s pose and state back to the STM32 microcontroller in real time, after which the PC analyzes and displays the data, and sends new control instructions to the microprocessor, which controls and adjusts the robot according to the target instructions.

The sensor part of perception layer is mainly composed of three kinds of sensors. The first is the posture angle measuring modules (JY901) that are equipped with MPU9250, which is mainly used to measure the bending angle of the module and the top of the internal soft bone. And the second is the membrane pressure sensors (IMS-C04A), which is used to measure the adsorption force generated when the suction cup adsorbs the wall. The last one is the three-dimensional Hall sensing module (CJMCU-90393) equipped with MLX-90393, which can be used to measure the displacement of the module and the internal soft bone. The sensors transmit the collected data to STM32 microcontroller through TTL (when controlling a single module) or IIC (when controlling multiple modules).

**Figure 11:**
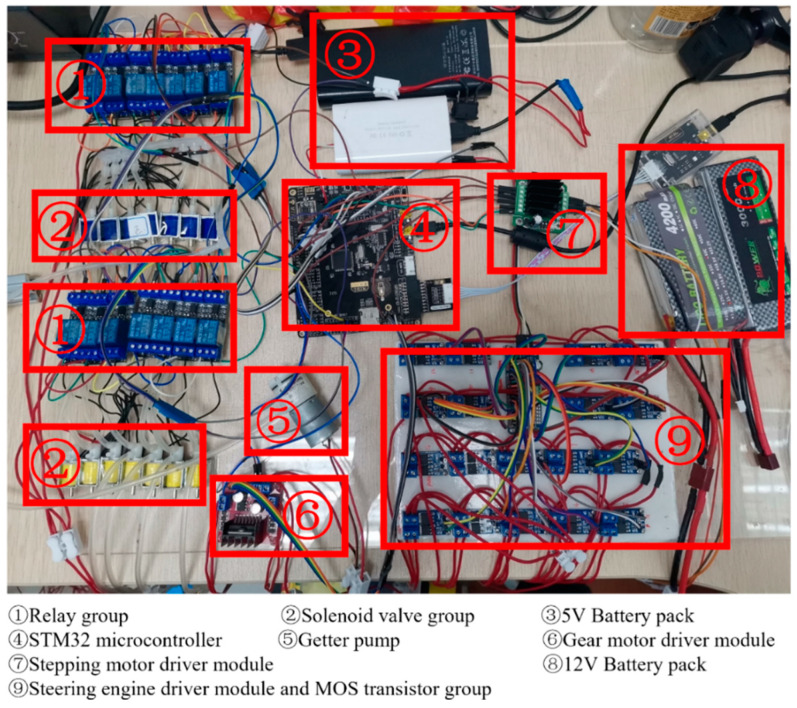
ISB-MWCR real machine test hardware system diagram.

According to the hierarchical principle of the control system in Figure 10, we have established a hardware system suitable for the robot, as shown in Figure 11. The hardware system includes a control section and driving device.

### 3.2. One-Way SMA Test Experiment

**Figure 12:**
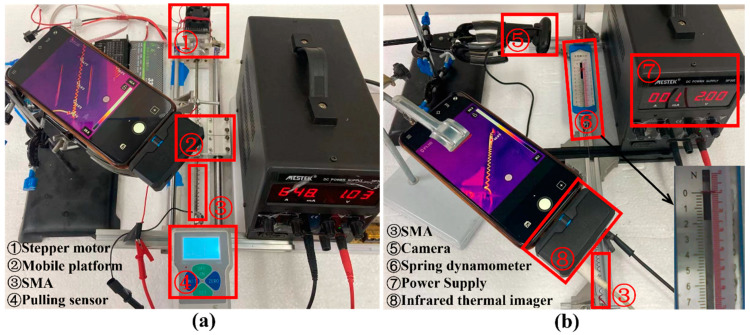
One-way SMA test platforms. (**a**) One-way SMA passive tensile test platform. (**b**) One-way SMA active shrinkage test platform.

The one-way SMA is a vital part of the ISB-MWCR. To obtain the expression of force F and time t at a specific voltage and the expression of force $F_{1}$, as shown in Figure 12, we designed two test platforms to test the passive stretch and active contraction of the one-way SMA.

**Figure 13:**
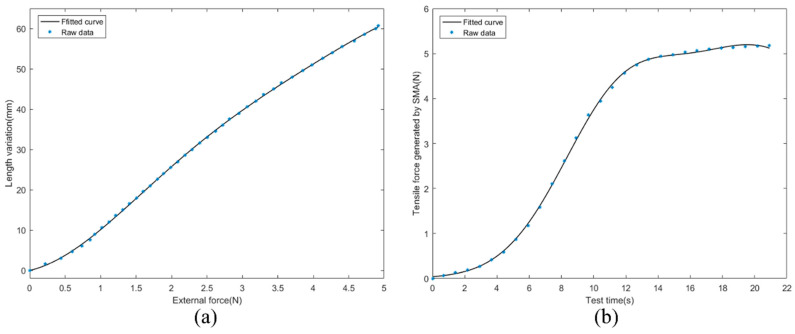
Passive tension and active contraction of the SMA. (**a**) When the SMA is passively stretched, the relationship between its tensile force and length change. (**b**) The relationship between the tensile force generated by SMA active contraction and the time when 2V voltage is applied at both ends of SMA.

In Figure 12a, one end of the SMA is affixed to the tension sensor, and the other end is affixed to the mobile platform controlled by the stepping motor, which is connected to the stepping motor by a screw rod on the slide rail. This device can adjust how far the SMA is stretched, adjust its initial length to $L_{s34}\approx 90\;\mathrm{mm}$, and make it so that the SMA produces no tension on the tension sensor. When the stepper motor directs the mobile platform to approach it, the SMA is elongated. The tensile force generated by the SMA (tensile force) and the step progress number of the stepper motor (which can be converted into the stretched amount of SMA) are then recorded. The SMA is in a state of power failure during stretching. The respective results of the test data are shown in Figure 13a.

In Figure 12b, one end of the SMA is affixed to the aluminum profile, and the other end is connected to the spring dynamometer by thin steel wire. If we adjust the length of the stretched SMA to $L_{s34}\approx 90\mathrm{mm}$, there is no tension on the spring dynamometer when the SMA is not powered on; however, the SMA will shrink when powered on. When a constant voltage of 2V is applied to the SMA, the SMA will contract. The tensile force (contraction force) generated by the SMA and the corresponding time are recorded. The respective results of the test data are shown in Figure 13b.

The curves in Figure 13a,b can be described as Equations (10) and (11), respectively:

(10) $$f\left(x\right)=a_{0}+a_{1}\cos\left(xw\right)+b_{1}\sin\left(xw\right)+a_{2}\cos\left(2xw\right)+b_{2}\sin\left(2xw\right)$$

(11) $$f_{1}{}_{\;}\left(x_{1}\right)=a_{3}e^{\left(-{\left(\frac{x_{1}-b_{3}}{c_{1}}\right)}^{2}\right)}+a_{4}e^{\left(-{\left(\frac{x_{1}-b_{4}}{c_{2}}\right)}^{2}\right)}$$

Their coefficients are recorded in Table 1. In Equation (10), f(x) is the length change after the one-way SMA is stretched, and x corresponds to the tensile force required to stretch a single SMA. Thus, there have:

(12) $$\{\begin{array}{c} f\left(x\right)=2\Delta L_{s34}\\ x=0.5F_{1} \end{array}\;$$

In Equation (11), $f_{1}\left(x\right)$ represents the tensile force generated when a single SMA shrinks, and $x_{1}$ represents the time t, where the combined tensile force $F=2f_{1}{}_{\;}\left(x_{1}\right)$. Thus far, the expressions of F and $F_{1}$ have been obtained, and the kinematics model of the bending robot module has been completed.

### 3.3. Exercise Experiment

To verify the correctness of motion planning and the feasibility of ISB-MWCR wall climbing, in this paper, experiments for climbing, steering, load movement, and span distance of ISB-MWCR are presented. The experiments can be seen in video (https://www.bilibili.com/video/bv1fq4y1V7w8 (accessed on 11 October 2021)). Refer to Table 2 for the mechanical structure parameters of the robot.

#### 3.3.1. Climbing Experiment

**Figure 14:**
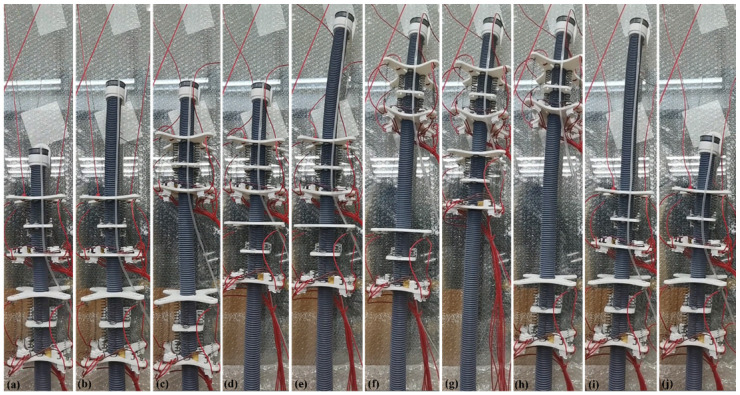
Experimental process of climbing and returning of two-module prototype on glass surface. (**a**–**j**) represents the motion of the robot.

The most basic movement of the wall-climbing robot is adsorption to the wall for climbing. We conducted an experiment that involved climbing up and turning along a glass surface using the ISB-MWCR two-module prototype, as shown in Figure 14. The experimental glass wall is installed at an angle of 80° to the ground, and the subsequent experimental wall is also a plane with an angle of 80°. The body length of a single module unit of the robot is about 110 mm. It can be seen from the figure that during the climbing process with the internal soft bone and module unit facing the glass (a–j represent the motion process of the robot), the maximum displacement step can reach 200 mm, which is about 1.8 times the length of the module. When moving down along the glass face, the maximum displacement step can reach 250 mm, which is about 2.3 times the length of the module.

#### 3.3.2. Steering Movement

In addition to analyzing the climbing movement, this study also carried out a series of steering experiments on the ISB-MWCR. The steering movement of the ISB-MWCR mainly relies on the contraction of one-way SMAs to bend the module, force the internal soft bone to bend, and finally, deflect the top of the robot to implement the steering movement. At the same time, the rotation of the top of the internal soft bone can also be used to offset the center of gravity to achieve the steering movement of the robot.

**Figure 15:**
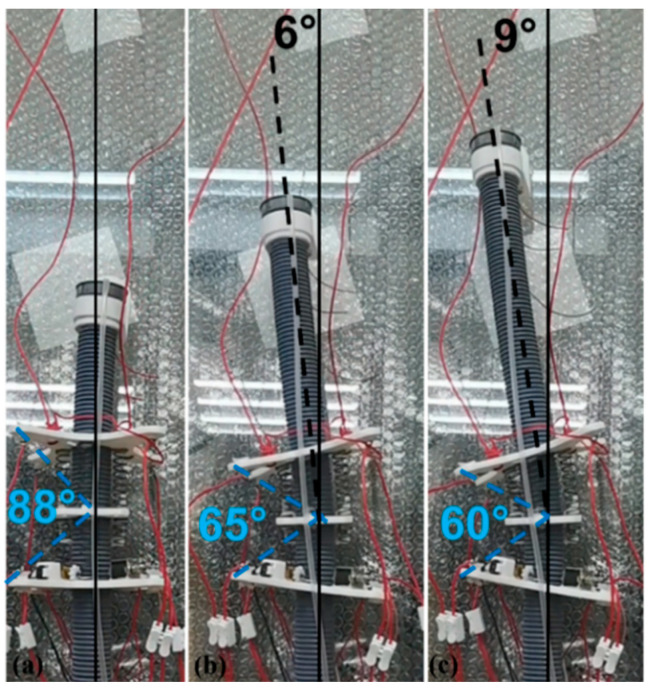
The process of robot attitude change during steering motion of single module prototype. (**a**–**c**) represents the motion of the robot.

First, we used the single module prototype to conduct a preliminary experiment on the bending effect of the robot, as shown in Figure 15. In the initial state, the top of the internal soft bone is hardly at an angle to the axis in the figure. The one-way SMA is also in the state of power failure and the angle between the upper and lower plates of the module is about 88°. The robot makes use of the bending of the module unit and the adjustment of the internal soft bones and the module unit position so that the front end of the robot has a deflection of about 9° to the left, and the angle between the upper and lower plates of the final module is about 60°.

**Figure 16:**
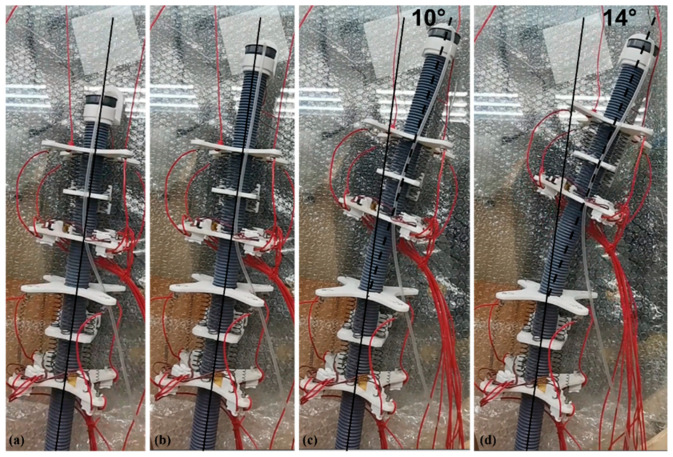
Two-module prototype offset to the right. (**a**–**d**) represents the motion of the robot.

Then, we prepared a two-module prototype and used it to test the offset effect that the ISB-MWCR can produce when it mainly depends on the center of gravity offset, as shown in Figure 16. The premise of the experiment is that the internal soft bone of the robot does not coincide with the direction of the center of gravity. The principle of this method is to extend the top of the internal soft bone and direct the stepping motor at the top to rotate to cause the top center of gravity to deviate from the original direction, to cause the robot to offset, and thus to enable the function of steering. As shown in Figure 16, the prototype has an offset of about 14° after a period of displacement.

**Figure 17:**
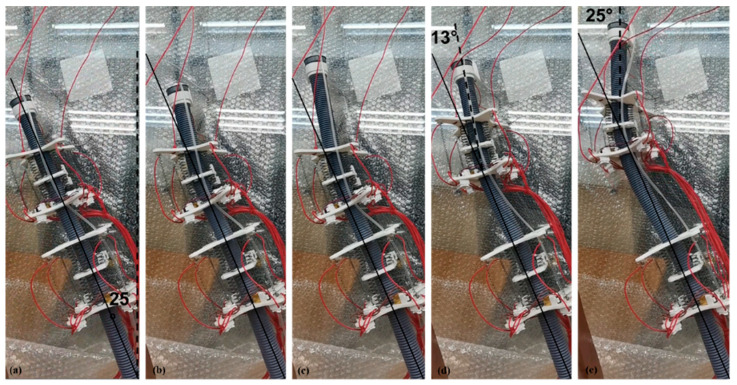
Two-module prototype used for tilt correction. (**a**–**e**) represents the motion of the robot.

Finally, after testing the deflection ability of the ISB-MWCR, we also designed a deflection-correction experiment, as shown in Figure 17. The experiment uses a two-module prototype for operation. At the initial stage, the body of the prototype is tilted about 25° and the internal soft bone is used as a reference. After motion adjustment, the robot straightened its upper body.

#### 3.3.3. Load Experiment

**Figure 18:**
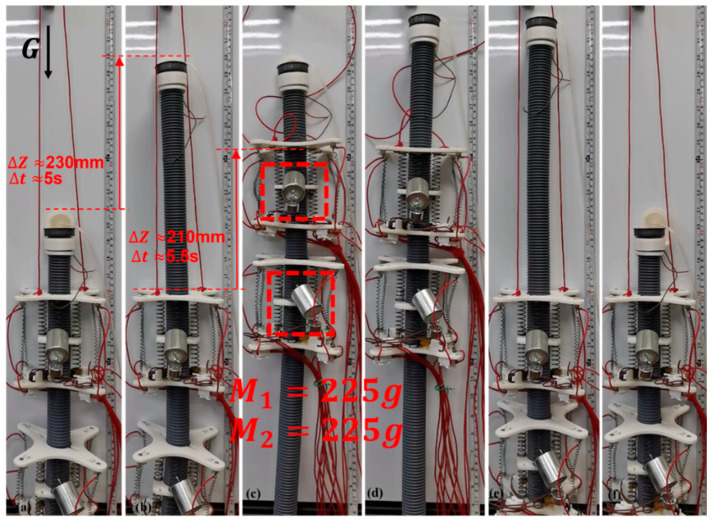
Two-module prototype load of 450 g (heavy object). (**a**–**f**) represents the motion of the robot.

Load capacity is an important performance index of wall-climbing robots. Therefore, this study first conducts a load test with a total weight of 450 g on the two-module prototype of ISB-MWCR, as shown in Figure 18. The total weight of the two-module prototype is about 700 g (excluding the wires connecting the robot); the weight of a single module is about 300 g, and the weight of the internal soft bone is about 100 g. In this experiment, (1) we carried 225 g weights on the two module units of the robot; (2) the load of the module is only 0.75 times the weight of the module itself; and (3) the uplink speed of the module can reach 38.2 mm/s.

**Figure 19:**
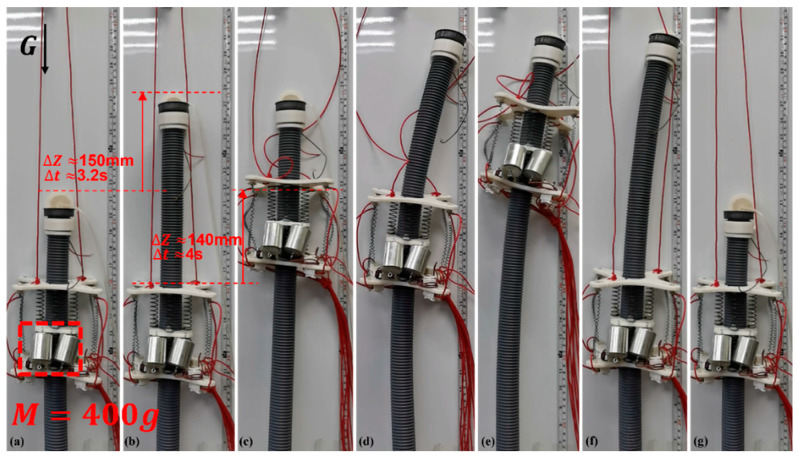
Maximum payload test of single module prototype. (**a**–**g**) represents the motion of the robot.

In the experiment, it was found that it is easy for a single module of the ISB-MWCR to carry a 225-g weight. To measure the maximum load capacity of a single module, we gradually increased the load size on the single module prototype. On the premise of ensuring the normal movement of the module, we increased the maximum load to 400 g, as shown in Figure 19. The payload of the module reached 1.33 times the module’s self-weight, and its uplink speed was about 35 mm/s.

#### 3.3.4. Spanning Experiment with Discontinuous Surface

**Figure 20:**
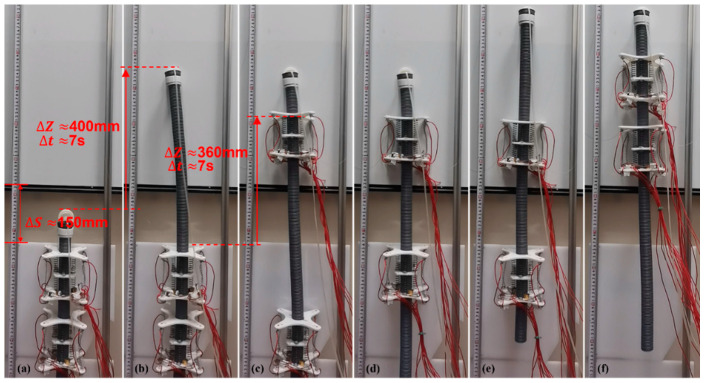
Two-module prototype spans discontinuous surface with large spacing. (**a**–**f**) represents the motion of the robot.

To test the adaptability of the ISB-MWCR to discontinuous walls, a discontinuous wall with adjustable spacing was designed in this study, as shown in Figure 20. The robot completed the crossing challenge of 150-mm spacing, which is about 1.37 times the length of the module (the body length of the robot module is 110 mm). The reason the robot is able to span such a relatively large distance is mainly due to the effective adsorption of the suction cup at the top of the robot, which benefits from the rigidity of the internal soft bone. When the robot transfers the internal soft bone upward, it is able to maintain stability so that the suction cup at the top can achieve good contact with the wall. The stable transmission distance is about 400 mm, which is about 3.6 times the length of the module. Therefore, the variable step distance of the robot is about 0 mm to 400 mm.

### 3.4. Payload Power Factor

To evaluate the load performance of the ISB-MWCR, it is convenient for scholars to compare. We propose a new performance metric for the load capacity of ISB-MWCR modules called the payload power factor (PPF). PPF refers to the driving unit of the ISB-MWCR under the same voltage and type. The ratio of the difference between the load power and the no-load power of the mobile module of the robot to the no-load power. The expression is defined as:

(13) $$W=\frac{P-K}{K}$$

*W* is the payload power factor; *P* is the load power, which is the product of the total weight of the load and the self-weight of the moving module and the average speed when the moving module moves upward; *K* is the no-load power, which is the product of the dead weight and the average speed of the moving module when it is unloaded and moving upwards. The units of *P* and *K* are N·m/s. PPF can be used to evaluate the load performance of the ISB-MWCR and also to reflect the utilization of driving force by the structure of the ISB-MWCR. The larger the value, the better the load performance of the robot and the higher the utilization of driving force in the structural design.

We will calculate the PPF of the ISB-MWCR. Because the load of the robot depends on the module, and the moving speed of the module is different from that of the internal soft bone, the PPF of the robot is scaled to a single module for calculation and description. According to Section 3.3.3, we conducted load and speed tests under three conditions. The results are shown in Table 3. The average speed is the average value of multiple tests.

The maximum payload of a single module of the ISB-MWCR is 400 g, and its corresponding PPF value is 0.582, so the maximum payload power factor of each robot module is 0.582.

## 4. Discussion

Single-module or two-module robots can perform basic climbing and steering movements. In the steering movement, the two-module robot was found to be more stable than the single-module robot and was able to make a complex attitude, as shown in the correction experiment in Figure 17. This action is impossible for a single module robot.

In the load experiment, the maximum payload of a single module of ISB-MWCR was 400 g (about 1.33 times the self-weight of the module). In theory, if the internal soft bones are long enough to allow each module to be directed upward and downward separately, the other modules will be adsorbed on the wall without allowing the heavy weight to cause plastic deformation inside the cartilage. Therefore, the robot can carry a virtually unlimited amount of weight, which demonstrates the variable load capacity of ISB-MWCR. When more and more modules are connected in series, the maximum weight of N modules are about 1.3 times that of the robot itself. Thus, the robot can greatly improve its load capacity by slightly increasing the complexity of the machine. According to the load performance index set in this paper, the maximum PPF of the robot module is 0.582.

In the experiment of crossing the discontinuous surface, the robot completed the crossing challenge of 150 mm (about 1.37 times the length of the module), and the maximum effective step distance reached 400 mm (about 3.6 times the length of the module). The experimental results show that it is feasible for the ISB-MWCR to span a discontinuous surface with large spacing. In the experiment, we also found that due to the characteristics of the internal soft bone materials, uncertain tilt sometimes occurs during movement. As shown in Figure 19, the top of the robot tilts in the process from e to g, which is caused by environmental interference. The flexibility of the internal soft bone makes it easy to deviate from the original motion direction due to external interference. In addition, the main factor affecting the maximum effective step distance of the robot is a material property of the internal soft bone, namely, its rigidity (the ability to maintain its original upright state). Another factor is the ability of the control system to adjust the posture of the internal soft bone. To this end, we can use a simple PID to adjust the attitude. To control the robot more accurately, more complex control algorithms can also be introduced, such as a boundary controller and a disturbance observer [27,28], which can reduce the uncertainty of flexible materials being affected by environmental disturbances, enhance the anti-interference adjustment ability of the robot during movement, and allow a greater breakthrough in the ability of the robot to span very large distances.

The purpose of this paper is to highlight the advantages of the structural design and motion mode of the robot in handling variable loads and deploying variable step distances. The robot utilizes suction cup adsorption and modular crawling motion. Due to the disadvantages of suction cup adsorption and crawling movement, the load of the robot is less than that of wall-climbing robots relying on grasping and magnetic adsorption. In terms of moving speed, it is far slower than wall-climbing robots using wheeled and tracked motion. However, the combination of these two adsorption and mobility modes provides significant advantages. The existing wall climbing robot can improve the load capacity by increasing the driving device or driving capacity, which usually greatly increases the complexity of the robot structure. By adding modules, ISB-MWCR slightly increases the complexity of the robot structure, but greatly improves the load capacity of the robot. Furthermore, the robot has the characteristic of variable step distance and can span a large distance on a discontinuous wall, which is difficult for other types of wall-climbing robots. Experiments demonstrated the load capacity of the robot and its ability to span a large distance.

## 5. Conclusions

In this study, a new bionic crawling modular wall-climbing robot based on internal soft bone was designed. The robot has variable load and variable step distance capability. The load capacity of this robot increases with the number of modules in series. The maximum load that *N* modules can carry is about 1.3 times the self-weight to allow movement. On the premise of stable movement, (1) the mobile walking distance of the robot can reach 3.6 times the length of the module; (2) it can span a discontinuous wall with 150 mm spacing; and (3) the effective variable step distance is 0 mm to 400 mm. In addition, we also proposed a performance index for the load performance of the ISB-MWCR’s modules, which is called the payload power factor. In the future, we will improve the adsorption created by the robot and implement an integrated design. The working environment of the robot will be modeled with a camera to plan the motion trajectory for the robot. Moreover, a trajectory-tracking controller [29] will be used to keep the robot system stable. Finally, to address the uncertain dynamics of the robot in the motion process, an adaptive fuzzy control scheme [30] will be adopted to track the desired trajectory.

## Figures and Tables

**Table 1:** Parameters of the respective function coefficients of SMA passive tensile and active shrinkage data.

Description	Value	Description	Value
$a_{0}$	34.58	$a_{3}$	5.066
$a_{1}$	−31.81	$b_{3}$	20.43
$b_{1}$	−2.051	$c_{1}$	8.616
$a_{2}$	−2.796	$a_{2}$	2.729
$b_{2}$	4.485	$b_{2}$	11.21
w	0.5904	$c_{2}$	5.08

**Table 2:** ISB-MWCR mechanical structure parameter table.

Description	Value
DC motor speed c	133 rpm
Gear tooth pitch b	4.5 mm
Number of gear teeth n	9
Distance from one-way SMA to central axis R	70 mm
Distance from spring to central axis r	24 mm
Spring coefficient k	0.835 N/mm
$\mathrm{Length}\;\mathrm{of}\;\mathrm{one}-\mathrm{way}\;\mathrm{SMA}\;\mathrm{after}\;\mathrm{stretching}\;L_{s34}$	90 mm
Body length of robot module	110 mm
Weight of robot module	300 g
Weight of internal soft bone	40 g
Weight of internal soft bone tip mechanism	60 g
Total weight of robot	700 g
Spring specification	1 × 12 × 50 mm
Thickness of upper, middle and lower plates	4 mm
Weight of upper and lower plates	25 g
Diagonal length of upper and lower plates	160 mm
Weight of middle plate	10 g
Side length of middle plate	60 mm
External diameter of internal soft bone	32 mm

**Table 3:** The no-load or load power of a module unit of the ISB-MWCR under three conditions.

Load Condition	Total Weight	Average Velocity	The No-Load or Load Power	PPF
No-load	3 N	0.051 m/s	1.53 N·m/s	----
Load 225 g	5.25 N	0.038 m/s	1.995 N·m/s	0.304
Load 400 g	7 N	0.035 m/s	2.42 N·m/s	0.582

## Data Availability

Not applicable.

## References

[1] Kanada A., Giardina F., Howison T., Mashimo T., Iida F. (2019). Reachability improvement of a climbing robot based on large deformations induced by tri-tube soft actuators. Soft Robot..

[2] Zhang H., Zhang J., Liu R. (2007). Mechanical design and dynamics of an autonomous climbing robot for elliptic half-shell cleaning. Int. J. Adv. Robot. Syst..

[3] Houxiang Z., Zhang J., Zong G., Wang W., Liu R. (2006). Sky Cleaner 3: A real pneumatic climbing robot for glass-wall cleaning. IEEE Robot. Autom. Mag..

[4] Zhang H.X., Zhang J.W., Zong G.H. Requirements of glass cleaning and development of climbing robot systems. Proceedings of the IEEE International Conference on Mechatronics & Automation.

[5] Sun D., Zhu J., Lai C., Tso S. (2004). A visual sensing application to a climbing cleaning robot on the glass surface. Mechatronics.

[6] Zhu J., Sun D., Tso S.-K. (2002). Development of a tracked climbing robot. J. Intell. Robot. Syst..

[7] Lee G., Kim H., Seo K., Kim J., Sitti M., Seo T. (2016). Series of multilinked caterpillar track-type climbing robots. J. Field Robot..

[8] Huang H., Li D., Xue Z., Chen X., Liu S., Leng J., Wei Y. (2017). Design and performance analysis of a tracked wall-climbing robot for ship inspection in shipbuilding. Ocean Eng..

[9] Eto H., Asada H.H. (2020). Development of a wheeled wall-climbing robot with a shape-adaptive magnetic adhesion mechanism. Proceedings of the 2020 IEEE International Conference on Robotics and Automation (ICRA).

[10] Garrido G.G., Sattar T.P. (2021). An autonomous wall climbing robot for inspection of reinforced concrete structures: SIRCAUR. J. Artif. Intell. Technol..

[11] Seriani S., Scalera L., Caruso M., Gasparetto A., Gallina P. (2019). Upside-down robots: Modeling and experimental validation of magnetic-adhesion mobile systems. Robotics.

[12] Nansai S., Elara M.R. (2016). A survey of wall climbing robots: Recent advances and challenges. Robotics.

[13] Bian S., Wei Y., Xu F., Kong D. (2021). A four-legged wall-climbing robot with spines and miniature setae array inspired by Longicorn and Gecko. J. Bionic Eng..

[14] Gao Y., Wei W., Wang X., Li Y., Wang D., Yu Q. (2021). Feasibility, planning and control of ground-wall transition for a suctorial hexapod robot. Appl. Intell..

[15] Khan M.B., Chuthong T., Do C.D., Thor M., Billeschou P., Larsen J.C., Manoonpong P. (2020). iCrawl: An inchworm-inspired crawling robot. IEEE Access.

[16] Jiang Q., Xu F. (2017). Grasping claws of bionic climbing robot for rough wall surface: Modeling and analysis. Appl. Sci..

[17] Lu Y., Zhou K., Ye N. (2017). Design and kinemics/dynamics analysis of a novel climbing robot with tri-planar limbs for remanufacturing. J. Mech. Sci. Technol..

[18] Guan Y., Zhu H., Wu W., Zhou X., Jiang L., Cai C., Zhang L., Zhang H. (2013). A modular biped wall-climbing robot with high mobility and manipulating function. IEEE/ASME Trans. Mechatron..

[19] Bian S., Xu F., Wei Y., Kong D. (2021). a novel type of wall-climbing robot with a gear transmission system arm and adhere mechanism inspired by Cicada and Gecko. Appl. Sci..

[20] Zhang W., Zhang W., Sun Z. (2021). A reconfigurable soft wall-climbing robot actuated by electromagnet. Int. J. Adv. Robot. Syst..

[21] Huang W., Xu Z., Xiao J., Hu W., Huang H., Zhou F. (2020). multimodal soft robot for complex environments using bionic omnidirectional bending actuator. IEEE Access.

[22] Cao J., Qin L., Liu J., Ren Q., Foo C.C., Wang H., Lee H.P., Zhu J. (2018). Untethered soft robot capable of stable locomotion using soft electrostatic actuators. Extreme Mech. Lett..

[23] Gu G., Zou J., Zhao R., Zhao X., Zhu X. (2018). Soft wall-climbing robots. Sci. Robot..

[24] Han I.H., Yi H., Song C.-W., Jeong H.E., Lee S.-Y. (2017). A miniaturized wall-climbing segment robot inspired by caterpillar locomotion. Bioinspiration Biomim..

[25] Qin L., Liang X., Huang H., Chui C.K., Yeow R.C.-H., Zhu J. (2019). A versatile soft crawling robot with rapid locomotion. Soft Robot..

[26] Huang W., Xiao J., Zeng F., Lu P., Lin G., Hu W., Lin X., Wu Y. (2021). A quadruped robot with three-dimensional flexible legs. Sensors.

[27] Zhao Z., He X., Ahn C.K. (2021). Boundary disturbance observer-based control of a vibrating single-link flexible manipulator. IEEE Trans. Syst. Man Cybern. Syst..

[28] Zhao Z., Liu Z. (2021). Finite-time convergence disturbance rejection control for a flexible Timoshenko manipulator. IEEE/CAA J. Autom. Sin..

[29] Yang C., Huang D., He W., Cheng L. (2021). Neural control of robot manipulators with trajectory tracking constraints and input saturation. IEEE Trans. Neural Netw. Learn. Syst..

[30] Yang C., Jiang Y., Na J., Li Z., Cheng L., Su C.-Y. (2019). Finite-time convergence adaptive fuzzy control for dual-arm robot with unknown kinematics and dynamics. IEEE Trans. Fuzzy Syst..

